# Supplementary material for: Towards real-world monitoring scenarios: An improved point prediction method for crowd counting based on contrastive learning
Source: PLoS One. 2025 Jul 2;20(7):e0327397. doi: 10.1371/journal.pone.0327397 (PMC12221087; doi:10.1371/journal.pone.0327397)
Supplement: Attachment — Submitted filename: Response to Reviewers.docx [file pone.0327397.s002.docx]

Dear Editors and Reviewers:

Thank you for your letter and for the reviewers’ comments concerning our manuscript entitled “Towards Real-world Monitoring Scenarios: An Improved Point Prediction Method for Crowd Counting Based on Contrastive Learning” (No.: PONE-D-24-51986). Those comments are all valuable and very helpful for revising and improving our paper, as well as the important guiding significance to our researches. We have studied comments carefully and have made correction which we hope meet with approval.

Reviewers' comments:

Reviewer's Responses to Questions

**1. Is the manuscript technically sound, and do the data support the conclusions?**

The manuscript must describe a technically sound piece of scientific research with data that supports the conclusions. Experiments must have been conducted rigorously, with appropriate controls, replication, and sample sizes. The conclusions must be drawn appropriately based on the data presented.

**Reviewer #1:** Partly

**Reviewer #2:** Partly

**The authors’ answer:**

We sincerely appreciate the reviewers for their acknowledgment of our paper. We have made every effort to improve the manuscript and have implemented several changes.

**2. Has the statistical analysis been performed appropriately and rigorously?**

**Reviewer #1:** Yes

**Reviewer #2:** No

**The authors’ answer:**

We sincerely appreciate the reviewers for their recognition of our paper. We have made every effort to improve the manuscript and have implemented several changes.

**3. Have the authors made all data underlying the findings in their manuscript fully available?**

The PLOS Data policy requires authors to make all data underlying the findings described in their manuscript fully available without restriction, with rare exception (please refer to the Data Availability Statement in the manuscript PDF file). The data should be provided as part of the manuscript or its supporting information, or deposited to a public repository. For example, in addition to summary statistics, the data points behind means, medians and variance measures should be available. If there are restrictions on publicly sharing data—e.g. participant privacy or use of data from a third party—those must be specified.

**Reviewer #1:** Yes

**Reviewer #2:** Yes

**The authors’ answer:**

We sincerely appreciate the reviewers for acknowledging our work.

4. **Is the manuscript presented in an intelligible fashion and written in standard English?**

PLOS ONE does not copyedit accepted manuscripts, so the language in submitted articles must be clear, correct, and unambiguous. Any typographical or grammatical errors should be corrected at revision, so please note any specific errors here.

**Reviewer #1:** Yes

**Reviewer #2:** No

**The authors’ answer:**

Thanks for your suggestion. We have made every effort to improve the manuscript and have implemented several changes. These modifications will not affect the content or structure of the paper.

**5. Review Comments to the Author**

**Reviewer #1:**

**1.The point prediction method mentioned in the paper has enhanced detection in both high- and low-density regions, but adaptability in complex scenarios is still a concern. Is the introduction of more training data or the use of data augmentation techniques considered to improve the robustness of the model? For example, training the model with different lighting conditions, weather changes, or different viewpoints may make it perform more robustly in real-world applications.**

**The authors’ answer:**

We have used more training data and data augmentation techniques in practical applications, but this part was not included in the paper to maintain consistency with other methods. Considering the reviewer's suggestion, we have added this part, which is highlighted in red in the Introduction section.

**2.How to further improve the detection accuracy in dense crowd scenes is an important research direction. The multi-scale feature fusion module mentioned in the paper is already a good attempt, but is it possible to explore other detection methods or optimize the existing framework? For example, it is possible to consider combining other deep learning models (e.g., the Transformer architecture) to enhance the feature extraction capability, or to introduce self-supervised learning methods to further improve the performance of the model.**

**The authors’ answer:**

It is possible to explore other detection methods or optimize the existing framework. Considering the reviewer's suggestion, we have added a discussion on this issue , which is highlighted in red in the Conclusion section.

**3. The model mentioned in the paper directly predicts header points during inference, which may affect real-time performance. Is it possible to optimize the computational efficiency of the model for real-time monitoring while maintaining accuracy?**

**The authors’ answer:**

The model we designed in the paper has already considered computational efficiency of the model for real-time monitoring. Our network is faster than other deep learning models such as Transformers. Besides, our method consumes less GPU memory, which makes practical applications more advantageous.

**4.It is mentioned in the paper that the method can be applied to the counting of other objects, such as vehicles and animals, in the future. Are there any plans to conduct relevant experiments to verify the effectiveness of the method in counting different objects? In addition, is it considered to combine the method with behavioral analysis to provide a more comprehensive monitoring solution?**

**The authors’ answer:**

Thanks for your suggestion. We have plans to conduct relevant experiments for counting different objects and combine the method with behavioral analysis in the future. However, we will not do the experiments in this paper due to the limitations of research conditions and article length.

Reviewer #2: This paper presents a novel approach to crowd counting using contrastive learning and point-based detection. While the work shows promise and addresses important challenges in crowd counting, several major issues need to be addressed.

**- The current experiments and analysis do not sufficiently demonstrate the contribution of contrastive learning. Please provide more thorough validation and ablation studies.**

**The authors’ answer:**

Thank you for your suggestion. We have already conducted an ablation study on the use of contrastive loss to demonstrate the contribution of contrastive learning. And we provide more thorough validation and ablation studies which is highlighted in red in the Qualitative Study on Contrastive learning part.

**- The paper organization needs significant improvement. Important technical details are scattered and sometimes unclear.**

**The authors’ answer:**

Thanks for your suggestion. We have revised the paper organization highlighted in red in the Our Method section.

**- Since the paper focuses on a real-world problem, the computational complexity analysis and comparison should be considered.**

**The authors’ answer:**

Thanks for your suggestion. We have added the computational complexity analysis and compared with other methods. This addition is marked in red in the Experiments section.

**- Please provide more detailed explanations and annotations for the figures (e.g., figure 1, 3, 6)**

**The authors’ answer:**

We have already provided explanations and annotations for the figures. Considering the reviewer's suggestion, we have added more explanations highlighted in red for the figures including figure 1, 3, 6.

**- Current literature reviews missed some recent works on local-global feature fusion and point/feature matching in visual learning, including [Transflow: Transformer as flow learner, 2023][Fusioncount: Efficient crowd counting via multiscale feature fusion, 2022][Lightglue: Local feature matching at light speed, 2023][Superthermal: Matching thermal as visible through thermal feature exploration, 2021], etc.**

**The authors’ answer:**

Thanks for your suggestion. We have revised the literature reviews highlighted in red in the Introduction and Related Work section.
