# Supplementary material for: Towards real-world monitoring scenarios: An improved point prediction method for crowd counting based on contrastive learning
Source: PLoS One. 2025 Jul 2;20(7):e0327397. doi: 10.1371/journal.pone.0327397 (PMC12221087; doi:10.1371/journal.pone.0327397)
Supplement: Attachment — Submitted filename: Response_to_Reviewers_auresp_2.docx [file pone.0327397.s003.docx]

Dear Editors and Reviewers:

Thank you for your letter and for the reviewers’ comments concerning our manuscript entitled “Towards Real-world Monitoring Scenarios: An Improved Point Prediction Method for Crowd Counting Based on Contrastive Learning” (No.: PONE-D-24-51986R1). Those comments are all valuable and very helpful for revising and improving our paper, as well as the important guiding significance to our researches. We have carefully studied the comments and made the necessary corrections, hoping that they will meet with your approval.

Reviewers' comments:

Reviewer's Responses to Questions

1. If the authors have adequately addressed your comments raised in a previous round of review and you feel that this manuscript is now acceptable for publication, you may indicate that here to bypass the “Comments to the Author” section, enter your conflict of interest statement in the “Confidential to Editor” section, and submit your "Accept" recommendation.

Reviewer #2: (No Response)

Reviewer #3: All comments have been addressed

**The authors’ answer:**

We sincerely appreciate the reviewers for their acknowledgment of our paper.

**2. Is the manuscript technically sound, and do the data support the conclusions?**

The manuscript must describe a technically sound piece of scientific research with data that supports the conclusions. Experiments must have been conducted rigorously, with appropriate controls, replication, and sample sizes. The conclusions must be drawn appropriately based on the data presented.

**Reviewer #2:** Partly

**Reviewer #3:**

**The authors’ answer:**

We sincerely appreciate the reviewers for their acknowledgment of our paper. We have made every effort to improve the manuscript and have implemented several changes.

**3. Has the statistical analysis been performed appropriately and rigorously?**

**Reviewer #2:** No

**Reviewer #3:**

**The authors’ answer:**

We sincerely appreciate the reviewers for their recognition of our paper. We have made every effort to improve the manuscript and have implemented several changes.

**4. Have the authors made all data underlying the findings in their manuscript fully available?**

The PLOS Data policy requires authors to make all data underlying the findings described in their manuscript fully available without restriction, with rare exception (please refer to the Data Availability Statement in the manuscript PDF file). The data should be provided as part of the manuscript or its supporting information, or deposited to a public repository. For example, in addition to summary statistics, the data points behind means, medians and variance measures should be available. If there are restrictions on publicly sharing data—e.g. participant privacy or use of data from a third party—those must be specified.

**Reviewer #2:** Yes

**Reviewer #3:**

**The authors’ answer:**

We sincerely appreciate the reviewers for acknowledging our work.

**5. Is the manuscript presented in an intelligible fashion and written in standard English?**

PLOS ONE does not copyedit accepted manuscripts, so the language in submitted articles must be clear, correct, and unambiguous. Any typographical or grammatical errors should be corrected at revision, so please note any specific errors here.

**Reviewer #2:** No

**Reviewer #3:**

**The authors’ answer:**

Thanks for your suggestion. We have made every effort to improve the manuscript and have implemented several changes. These modifications will not affect the content or structure of the paper.

**6. Review Comments to the Author**

**Reviewer #2:**

1. Some captions for the figures are too simple, the authors should provide an indepth analysis for each figure. For example, while the Fig. 6 illustrates the impact of different patch numbers on MAE, the caption should further explain key trends, such as why the MAE fluctuates after a certain threshold and how this finding supports the proposed method.

**The authors’ answer:**

Thanks for your suggestion. We have revised the illustration, highlighted in red, for explaining key trends in MAE of different patch numbers in the Ablation Experiments section.

2. In Figure 1, the point-based detection results are really hard to recognize. The authors may consider changing the point color.

**The authors’ answer:**

Thanks for your suggestion. We have changed the point color and point size to make the point-based results easier to recognize.

3. Some sections could be better structured for clarity, particularly in the explanation of contrastive learning.

**The authors’ answer:**

Thanks for your suggestion. We have modified the paper structure highlighted in red in the Our Method section. In the section on contrastive learning network design and loss function design, we elucidate the methodology of leveraging contrastive learning to enhance the performance of crowd counting.

**4. I recommend a thorough revision of the writing to improve readability.**

**The authors’ answer:**

Thanks for your suggestion. We have made every effort to improve the manuscript and hope to meet with approval.

Reviewer #3: Thank you for taking the time to consider and thoughtfully address my comments. I appreciate your attention to detail and your commitment to incorporating constructive feedback to improve the overall quality of the work

**The authors’ answer:**

We sincerely appreciate your acknowledgment of our paper.
